# Spatial prioritization of dugong habitats in India can contribute towards achieving the 30 × 30 global biodiversity target

**DOI:** 10.1038/s41598-024-64760-8

**Published:** 2024-06-17

**Authors:** Sohom Seal, Sharad Bayyana, Anant Pande, Chinmaya Ghanekar, Prachi Sachchidanand Hatkar, Sameeha Pathan, Shivani Patel, Sagar Rajpurkar, Sumit Prajapati, Swapnali Gole, Sweta Iyer, Aditi Nair, Nehru Prabakaran, Kuppusamy Sivakumar, Jeyaraj Antony Johnson

**Affiliations:** 1 Department of Habitat Ecology, Wildlife Institute of India, P.O. Chandrabani, Dehradun, Uttarakhand 248 001 India; 2 Centre for Biodiversity and Conservation Science, School of Environment, University of Queensland, St. Lucia, QLD 4072 Australia; 3 Marine Program, Wildlife Conservation Society - India, Bengaluru, Karnataka 560 097 India; 4 Department of Ecology and Environment, Pondicherry University, Puducherry, India

**Keywords:** Species distribution modelling, Environmental niche modelling, Marine mammal conservation, Habitat risk assessment, Marine spatial planning, Ecology, Conservation biology, Ecological modelling, Ocean sciences, Marine biology, Ecology, Conservation biology, Ecological modelling

## Abstract

Indian coastal waters are critical for dugong populations in the western Indian Ocean. Systematic spatial planning of dugong habitats can help to achieve biodiversity conservation and area-based protection targets in the region. In this study, we employed environmental niche modelling to predict suitable dugong habitats and identify influencing factors along its entire distribution range in Indian waters. We examined data on fishing pressures collected through systematic interview surveys, citizen-science data, and field surveys to demarcate dugong habitats with varying risks. Seagrass presence was the primary factor in determining dugong habitat suitability across the study sites. Other variables such as depth, bathymetric slope, and Euclidean distance from the shore were significant factors, particularly in predicting seasonal suitability. Predicted suitable habitats showed a remarkable shift from pre-monsoon in Palk Bay to post-monsoon in the Gulf of Mannar, indicating the potential of seasonal dugong movement. The entire coastline along the Palk Bay-Gulf of Mannar region was observed to be at high to moderate risk, including the Gulf of Mannar Marine National Park, a high-risk area. The Andaman Islands exhibited high suitability during pre- and post-monsoon season, whereas the Nicobar Islands were highly suitable for monsoon season. Risk assessment of modelled suitable areas revealed that < 15% of high-risk areas across Andaman and Nicobar Islands and Palk Bay and Gulf of Mannar, Tamil Nadu, fall within the existing protected areas. A few offshore reef islands are identified under high-risk zones in the Gulf of Kutch, Gujarat. We highlight the utility of citizen science and secondary data in performing large-scale spatial ecological analysis. Overall, identifying synoptic scale ‘Critical Dugong Habitats’ has positive implications for the country's progress towards achieving the global 30 × 30 target through systematic conservation planning.

## Introduction

Marine mammals are known to benefit from effective area-based conservation measures such as Marine Protected Areas (MPAs)^1^. Given the migratory nature of these species, delineating MPAs through effective spatial planning in an ecosystem context is strongly advised^2^. India recently declared its first marine mammal-focused Marine Protected Area (MPA) in the country^3^. About 448 sq. km area was notified as Dugong Conservation Reserve, adjoining Thanjavur and Pudukkottai districts of Tamil Nadu state, bordering the south-east coast of India in the Palk Bay region. This newest MPA in the country aims to protect globally vulnerable but regionally endangered populations of primarily herbivorous marine mammals—dugongs, that occur in disjunct pockets^4^ and are declining rapidly from most of their range^5,6^. Reasons for their decline are attributed to degradation of seagrass habitats, loss of reproductive potential and increasing anthropic pressures across their distribution^7–9^. With large populations reported from the extreme ends of their global geographical distribution (the east coast of Australia extending up to New Caledonia and the east coast of Africa)^4,10–12^, the dugong population in India are the major hope for the species’ persistence in South Asia.

Indian dugong populations exist within large MPAs such as the Gulf of Kutch Marine National Park (MNP), Gulf of Mannar MNP, Rani Jhansi MNP and Mahatma Gandhi MNP as well as in the waters beyond MPA boundaries^13^. Expanding the area coverage with effective management of these MPAs would help in halting the population decline, reducing disturbances, retaining seagrass ecosystems, and ensuring the long-term survival of dugongs. As of now, MPAs recognized through legislation span only 0.26% of India's geographical area, falling drastically short of the 10% area-based protection for coastal and marine areas envisaged in the Aichi Biodiversity Target 11 (Strategic Plan for Biodiversity 2011–2020). Overall, MPA cover (8717 Km^2^) is less than 5% of the overall Protected Area (PA) cover (178,640 Km^2^) which includes terrestrial PAs^14^. In the wake of the adoption of the Kunming-Montreal Global Biodiversity Framework (henceforth GBF;^15^), which requires signatory countries to conserve at least 30% of their terrestrial, inland water, coastal, and marine areas by 2030 (Target 3, also known as '30 × 30'), effective area-based conservation measures for coastal and marine areas in the country is an urgent priority. Action-oriented targets such as '30 × 30' can be complemented with biodiversity outcome-focused conservation^16^, such as protecting and restoring seagrass habitats to facilitate dugong movement, increase the species' genetic diversity, and improve ecosystem functioning.

Understanding species distribution is crucial to identifying important habitats and formulating effective conservation strategies for long-term preservation^17^. Dugong distribution and occupancy are dependent upon various biological (such as seagrass availability^7,18^), environmental (sea surface temperature^19^; turbidity, sea state^20^) and topographic (depth^21^ parameters). In India, dugong occupancy has declined substantially from Indian territorial waters (over 85% in some parts^22^). In some isolated regions, such as the tropical oceanic islands of Andaman and Nicobar Islands, a 60% reduction in occupancy was observed in the last few decades^13^. With the increasing human footprint in the ocean, the rise in activities such as vessel traffic and overfishing (Supplementary Figs. S1, S2 online) in seagrass habitats^23^ are presumed to have deleterious impacts on dugong distribution. Also, mechanized and motorized vessels comprise > 99% of all the registered vessels in two of the targeted study states^24^, risking the dugong population to a great extent. Currently, the extent of these impacts on dugongs in India is poorly known but is apparently deduced through high incidences of strandings^25,26^.

Researchers often rely upon presence‐only data when systematic occurrence data on certain species is unavailable (i.e., when species absence is not verified). These uncertainties in species occurrence data stem from opportunistic and sometimes unstructured sampling^27^. Recent advances in species distribution modelling (SDM) or ecological niche modelling (ENM) methods, such as MaxEnt^28^, are significant due to their utility in the analysis of presence‐only data through user‐friendly algorithms^29^. These methods reduce effort and cost for large spatial scale species monitoring programmes^30,31^. In the past few years, SDM has been increasingly favoured by numerous research groups engaged in wildlife monitoring^31^. This approach has also been influential in developing conservation strategies for marine mammals^32–34^.

In the case of dugongs, identifying critical habitats is enormously significant for seascape-level planning and optimal usage of management resources^13,35^. In the present study, we utilized a comprehensive dataset generated through multiple approaches (semi-structured questionnaires, citizen science, volunteer networks, and primary field surveys)^11^. We predict highly suitable dugong habitats within their current area of distribution in Indian coastal waters, identify high-risk areas, and validate them with primary surveys to classify them as Critical Dugong Habitats (CDHs). In the wake of the '30 × 30' target and National Biodiversity Targets^36^, we highlight the importance of area-based conservation of CDHs to inform dugong conservation with recommendations for site-specific management interventions.

## Results

### Seagrass distribution

**Figure 1:**
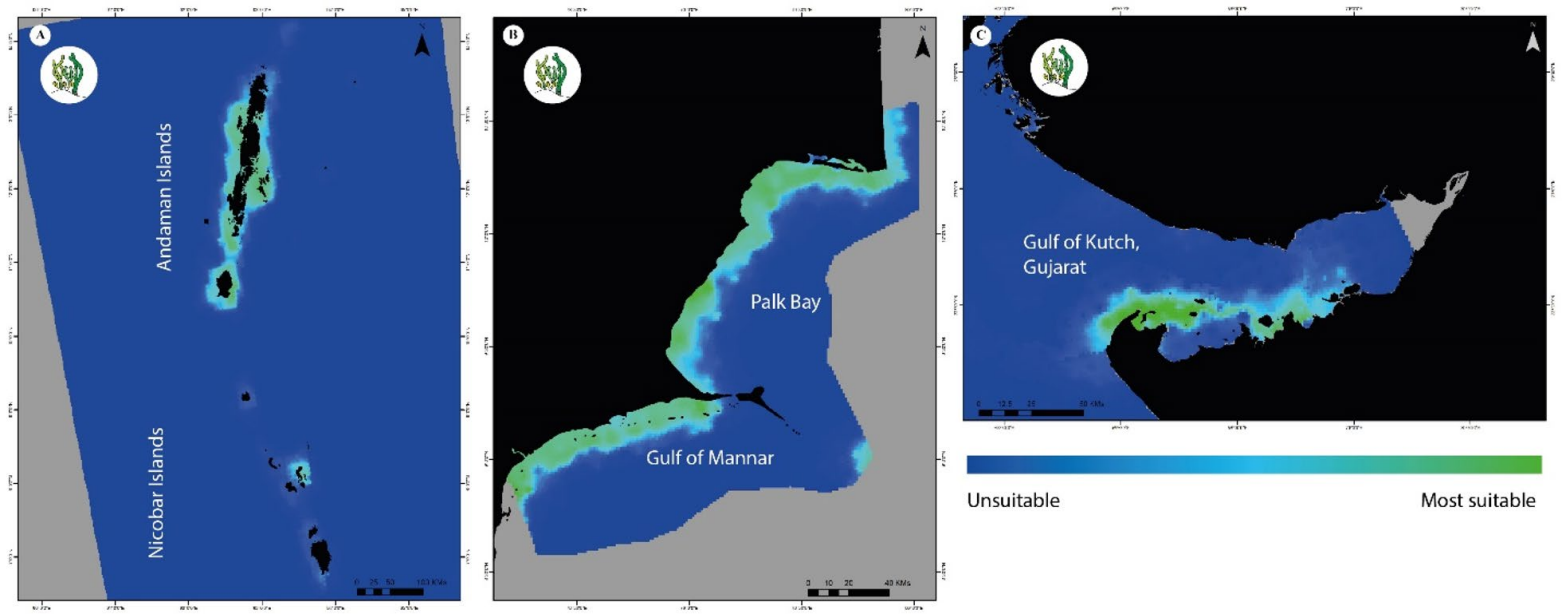
Habitat suitability for seagrass meadows in (**A**) Andaman and Nicobar Islands (**B**) Palk Bay and Gulf of Mannar, Tamil Nadu and (**C**) Gulf of Kutch, Gujarat. Here, ‘unsuitable’ corresponds to 0 and ‘most suitable’ corresponds to 1. The suitability layers were generated using MaxEnt v.3.4.4 software (https://biodiversityinformatics.amnh.org/open_source/maxent), and the final maps were generated using ArcGIS Pro v.3.2.1 software (https://www.esri.com/en-us/arcgis/products/arcgis-pro/overview).

Seagrass distribution was predicted along the entire Andaman Islands group with specific patches around central Nicobar (Camorta and Trinket) and Great Nicobar with AUC ± SD = 0.924 ± 0.002, indicating the accuracy of the model (Fig. 1).

The bathymetric depth and wave height had a maximum contribution (84.3%) toward seagrass prediction in this region (Supplementary TableS3 online).

Slope, salinity, photosynthetically active radiation (PAR), pH and sea surface temperature (SST) were the least contributing factors in the ANI region. In the PB-GoM region, maximum sea surface temperature and distance from the shore showed an 83.9% contribution (Supplementary Table S3 online). The entire PB-GoM coast was predicted for seagrass presence, with north Palk Bay and the north GoM showing maximum probability (AUC ± SD = 0.850 ± 0.004). Whereas, in the case of GoK, seagrass was strongly predicted in the southern part of the gulf around the islands situated in the region (AUC ± SD = 0.952 ± 0.058) (Fig. 1). The availability of phosphate and mean sea surface temperature were key contributing factors (67%) in this region.

### Seasonal dugong distribution

#### Andaman and Nicobar islands (ANI)

**Figure 2:**
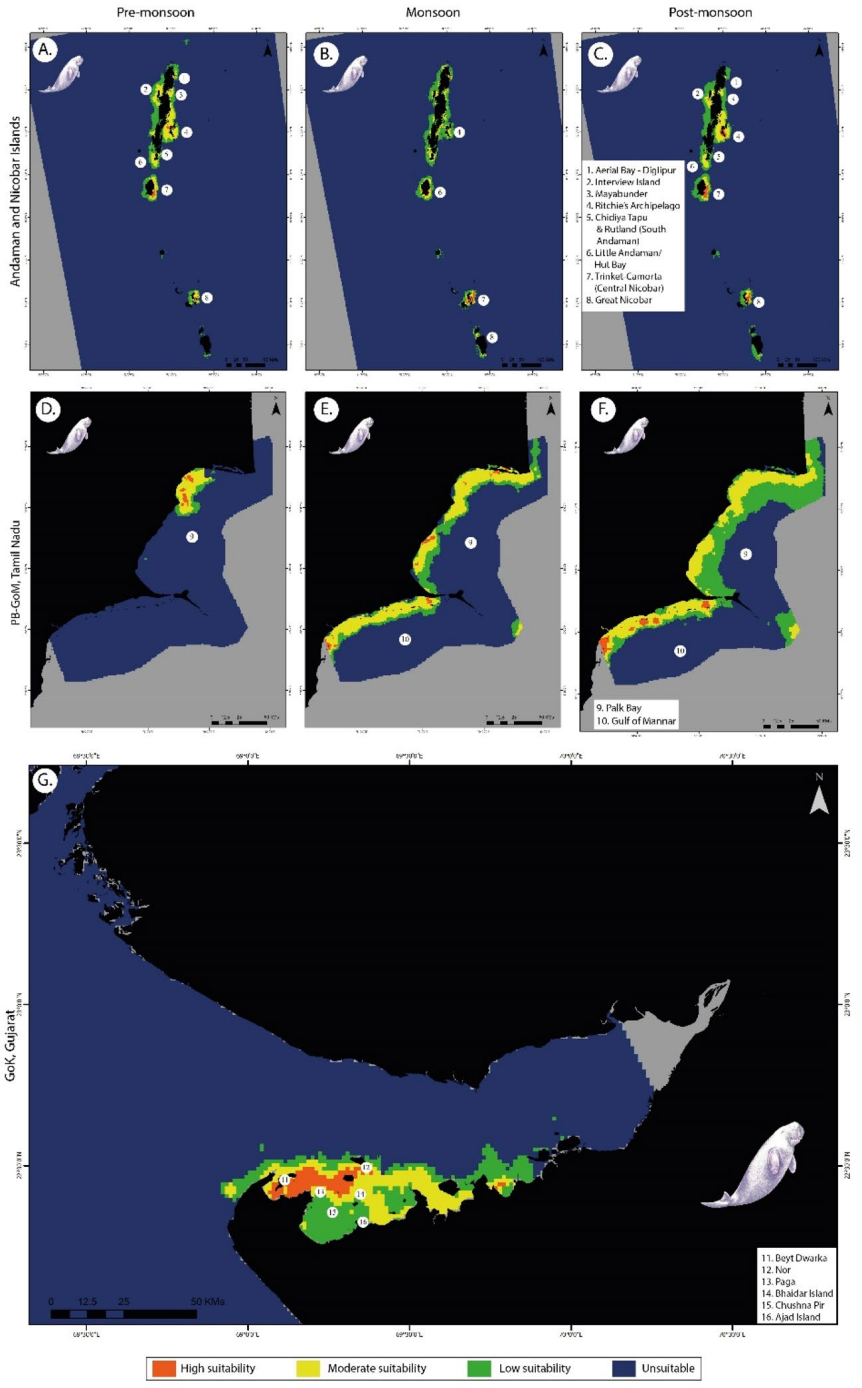
Habitat suitability predictions for dugongs in (**A**) pre-monsoon, (**B**) monsoon, (**C**) post-monsoon at Andaman & Nicobar Islands; (**D**) pre-monsoon, (**E**) monsoon, (**F**) post-monsoon at Palk Bay and Gulf of Mannar, Tamil Nadu; and (**G**) Gulf of Kutch region, Gujarat. Here, each class corresponds to values 0–0.25 (unsuitable), 0.25–0.5 (low suitability), 0.5–0.75 (moderately suitable) and 0.75–1 (highly suitable) regions, respectively. The suitability layers were generated using MaxEnt v.3.4.4 software (https://biodiversityinformatics.amnh.org/open_source/maxent), and the final maps were generated using ArcGIS Pro v.3.2.1 software (https://www.esri.com/en-us/arcgis/products/arcgis-pro/overview).

Ritchie's archipelago, the Hut Bay region of Little Andaman, and the region around Trinket-Camorta islands in central Nicobar were identified as highly suitable regions for all seasons. Aerial Bay, Diglipur, Mayabunder in the north, Interview Island in the west and Rutland & Chidiya Tapu regions of south Andaman were recorded as moderately suitable regions in the pre- and post-monsoon season (Fig. 2).

During the monsoon season, highly suitable areas are more prominent in Central and Great Nicobar Island than in the other two seasons. High suitable areas across Andaman have spread out during the monsoon season, whereas it is restricted to Little Andaman and Ritchie's Archipelago during pre- and post-monsoon seasons. The model performances were noted across seasons for the ANI region with AUC ± SD = 0.987 ± 0.003 (pre-monsoon), AUC ± SD = 0.987 ± 0.005 (monsoon), AUC ± SD = 0.982 ± 0.008 (post-monsoon). The statistical correlation matrix performed among the seasons showed a high correlation, indicating that habitat suitability was similar (Supplementary Table S4 online).

#### Palk Bay and Gulf of Mannar (PB-GoM)

In the PB-GoM region, there were visible changes in the suitable habitats between the seasons. During the pre-monsoon season, high habitat suitability was observed in the north Palk Bay region, whereas the rest of the region was categorized as low to unsuitable habitats (Fig. 2). This eventually diffused along the entire stretch with the onset of retreating monsoon, predicting patches of high suitable region along moderate to low suitable habitats which further concentrated high suitable regions in the GoM region, post-monsoon. The predictive power of the seasonal MaxEnt models for this region was assessed with AUC ± SD = 0.96 ± 0.029 (pre-monsoon), AUC ± SD = 0.911 ± 0.022 (monsoon), AUC ± SD = 0.825 ± 0.031 (post-monsoon). Though the extent of highly suitable habitats is significantly lower in comparison to moderate and low suitable regions, there is a noticeable trend in the shift of suitable habitats from north to south from pre-monsoon to post-monsoon seasons. This was further supported with I-statistics correlation analysis (Supplementary Table S4 online), where there is a significant difference between pre-monsoon and post-monsoon site suitability estimates.

#### Gulf of Kutch (GoK)

Annual habitat suitability output of the GoK region showed high suitability near Paga, Bhaidar and Chushna Pir extending up to Beyt Dwaraka island (Fig. 2). Further, a moderately suitable habitat region extending from Beyt Dwarka island and beyond Nor Island in the East is identified. Subtidal regions southwards to Chushna Pir, including Ajad Island, were identified as having low suitable habitats for dugongs throughout the year in this region. The averaged AUC value obtained from MaxEnt models was 0.983 (± 0.005).

#### Variable contributions

We found seagrass presence to be the major factor in determining suitable dugong habitats along the study areas. In the ANI region, seagrass contribution was more than 50% for all three seasons, fluctuating from > 70% in the pre-monsoon and post-monsoon to 58% during the monsoon. Followed by seagrass, the contribution of depth as a variable for site suitability was approximately 1/5th in all three seasons. Further, distance from shore, slope and diffuse attenuation coefficient significantly contributed to suitability prediction in the ANI region (Table 1, Supplementary Fig. S5 online). In PB-GoM, we observed that the seagrass contribution doubled (33.5–70.8%) from pre-monsoon to post-monsoon season (Table 1). The slope was a variable that contributed more than 50% in the pre-monsoon season, followed by seagrass presence at 33.5%. Sea Surface Temperature (SST) proved significant during the monsoon season, whereas Slope, Depth, and Mean SST contributed equally during the post-monsoon season in the PB-GoM region. Depth, distance from the shore and diffuse attenuation coefficient (DAC) had a noticeable impact on the PB-GoM region for all three seasons. For GoK, with a 70% variable contribution of seagrass presence, the depth variable followed with nearly 20% contribution. Lastly, Diffuse Attenuation Coefficient, Distance from the shore and minimum SST contributed equally ~ 2.5%.

**Table 1:** List of variables finalized for modelling dugong distribution in pre-monsoon, monsoon and post-monsoon in Andaman and Nicobar Islands (ANI), Palk Bay & Gulf of Mannar (PB-GoM) of Tamil Nadu, and Gulf of Kutch (GoK) of Gujarat along with their contribution percentages.

Layers	ANI			PB-GoM			GoK
	Pre Monsoon	Monsoon	Post Monsoon	Pre Monsoon	Monsoon	Post Monsoon	
Seagrass presence^$^	76.2	58.3	73.6	33.5	66	70.8	70.5
Depth (m)*	14.8	18.9	18.2	8.8	2.2	5.7	20.3
Slope (°)*	2.1	1.9	3.8	51.4	0.3	5	0.6
Distance from the shore (km)*	5.3	12.5	4.2	0.9	5.3	1.8	2.8
Monthly diffuse attenuation coefficient (Kd)#	0.2	7.2	0	5	3.3	11	2.4
Monthly sea surface temperature-mean (°C)^#^	1.4	1.2	0.1	0.5	23	5.6	0.8
Monthly sea surface temperature-max (°C)^#^	–	–	–	–	–	–	0.4
Monthly sea surface temperature-min (°C)^#^	–	–	–	–	–	–	2.2

‘–’ not used; Sources: *Global Marine Environmental Datasets (GMED)^37^; ^#^NASA Ocean Colour; ^$^Modelled data (MaxEnt).

### Risk assessment

**Figure 3:**
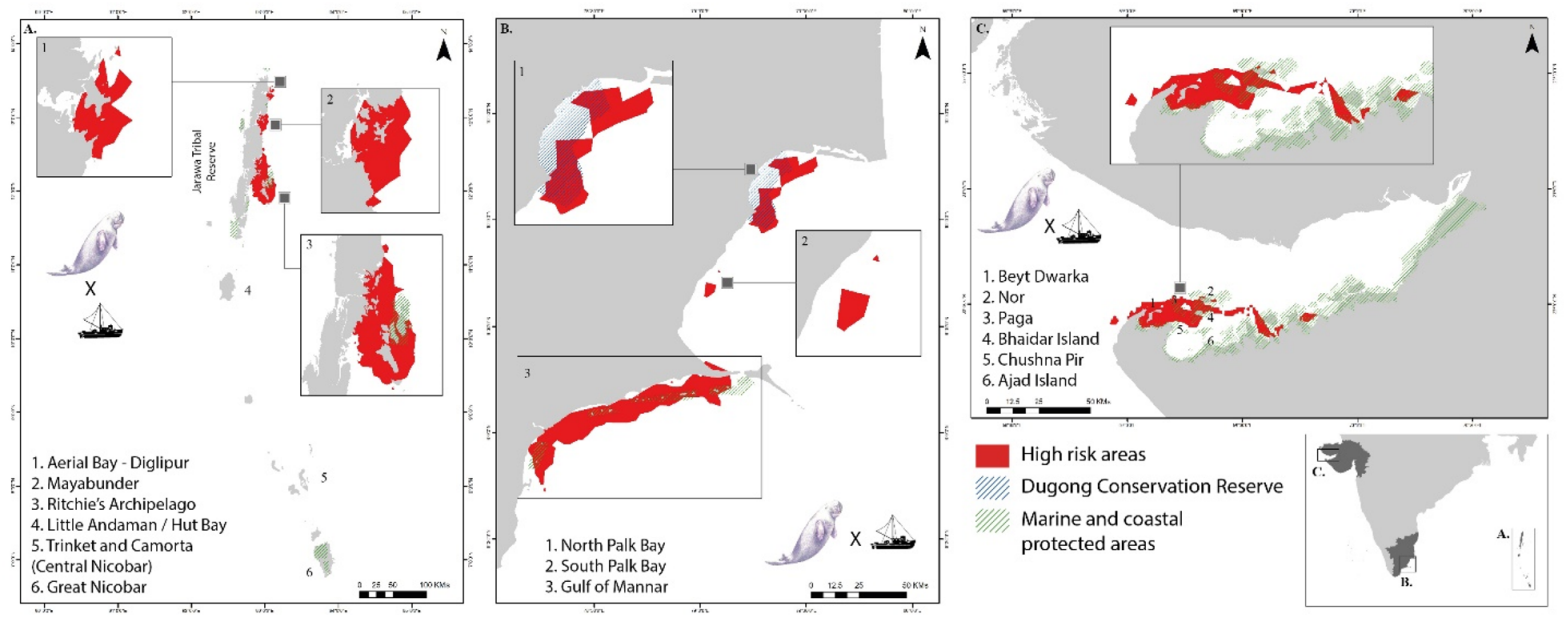
Risk predictions due to fishing pressure in (**A**) Andaman and Nicobar Islands; (**B**) Palk Bay and Gulf of Mannar, Tamil Nadu and (**C**) Gulf of Kutch, Gujarat. Here, the high-risk class corresponds to 50% and above values of the total range, overlaid with current marine protected areas. The output maps were generated using ArcGIS Pro 3.2.1 software (https://www.esri.com/en-us/arcgis/products/arcgis-pro/overview).

A singular consolidated risk assessment was conducted for all three regions by averaging the seasonal habitat suitability predictions for all seasons. Highly suitable habitats were also at a higher risk due to anthropogenic influences in all three regions (Fig. 3).

**Figure 4:**
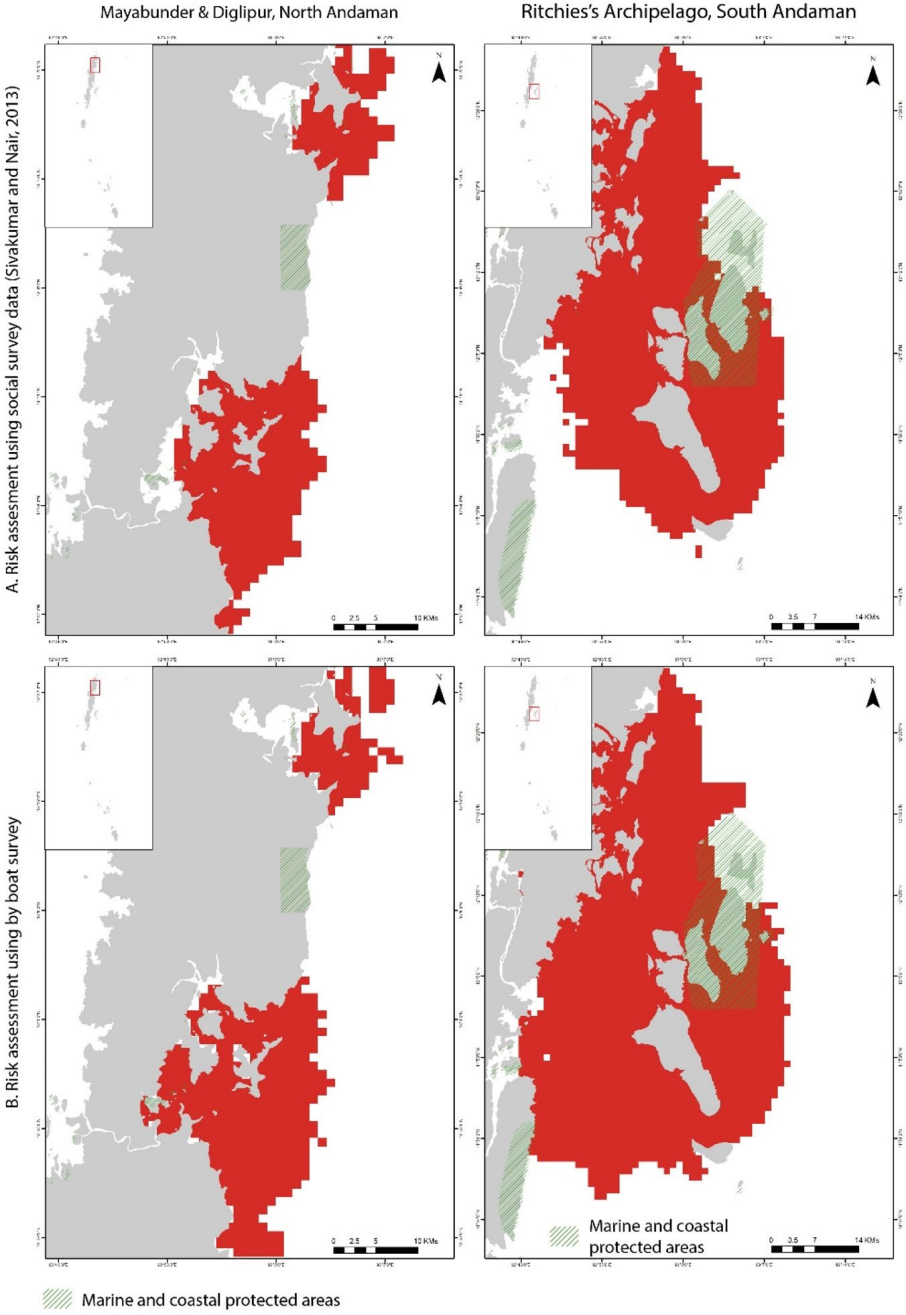
Comparison of selected high-risk habitats. The top row shows the risk based on suitability models derived from social surveys, and the bottom row shows the result from the boat-based surveys [Mayabunder and Diglipur, North Andaman (left) and Ritchie's Archipelago, South Andaman (right)]. The output maps were generated using ArcGIS Pro 3.2.1 software (https://www.esri.com/en-us/arcgis/products/arcgis-pro/overview).

In the ANI region, the risk is high in specific locations along the Andaman group of islands. Ritchie's archipelago, Aerial Bay, and Mayabunder region show high risk, with almost the entire coast susceptible to moderate risk due to human activities, except the west coast in the Middle Andaman region (Supplementary Fig. S6 online). Risk assessment using boat-based surveys confirmed the high-risk region in Aerial Bay-Diglipur and in the Mayabunder region (Fig. 4).

The Rani Jhansi Marine National Park falls under the high-risk zone in the risk assessment analysis that extends up to middle Andaman to Long Island (Fig. 4). Similarly, the entire coast of Little Andaman is predicted for moderate amount of risk for the dugong population. A moderate threat level is available in the Nicobar region, mainly concentrated around Trinket and Camorta Islands in the Middle Nicobar region and the southern coast of Great Nicobar Island (Supplementary Fig. S6 online).

**Figure 5:**
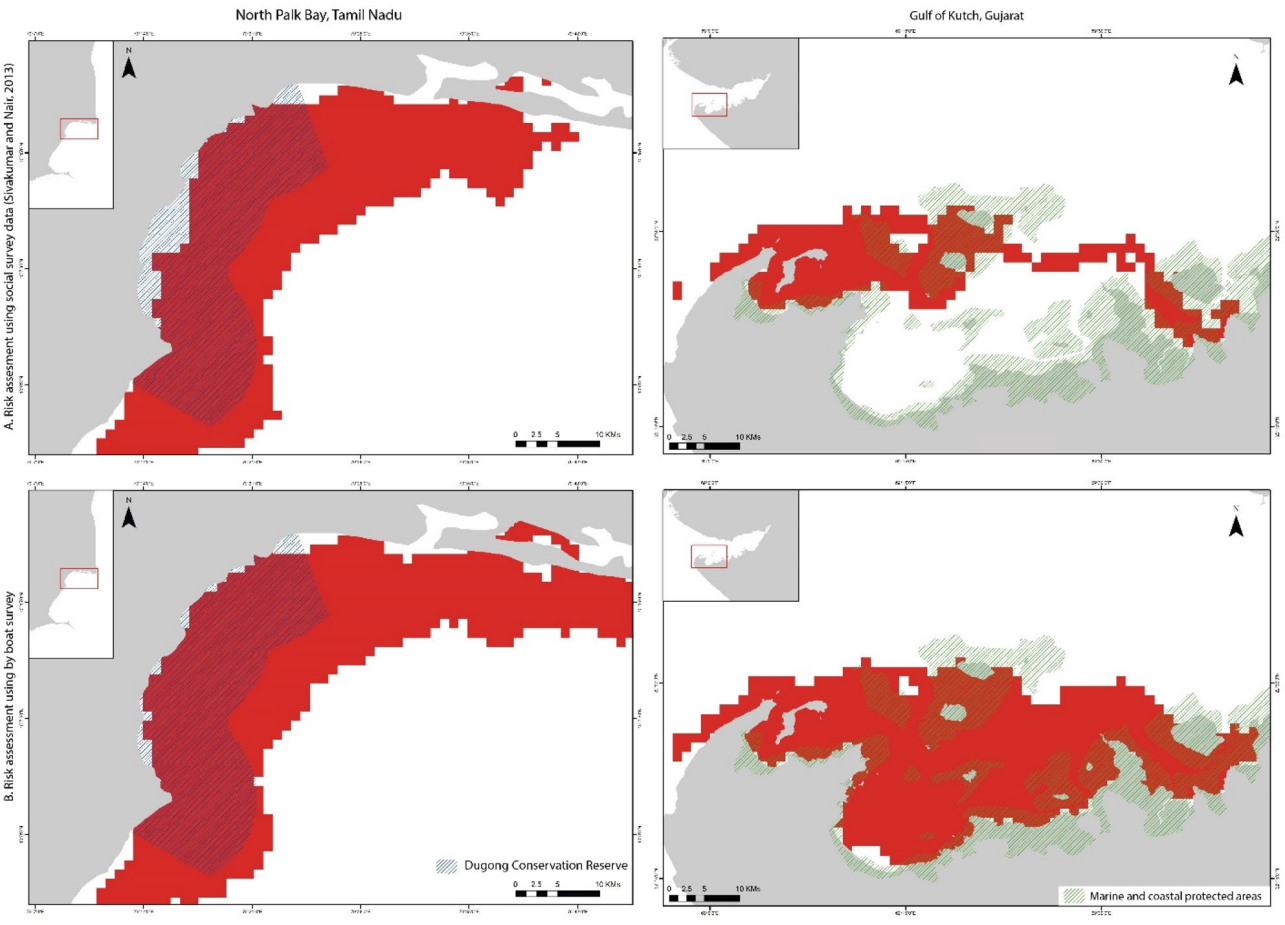
Comparison of selected high-risk habitats. The top row shows the risk based on suitability models derived from social surveys, and the bottom row shows the result from the boat-based surveys [North Palk Bay, Tamil Nadu (left) and Gulf of Kutch, Gujarat (right)]. The output maps were generated using ArcGIS Pro 3.2.1 software (https://www.esri.com/en-us/arcgis/products/arcgis-pro/overview).

In the PB-GoM, the extent of high-risk zones is more than 50% of the entire region under prediction. Boat-based risk assessment provided similar observations with a precise prediction for the east and northeast regions in North Palk Bay (Fig. 5).

Further, the entire coast in the study area is under moderate to high risk (Supplementary Fig. S6 online), indicating severe anthropogenic pressure (here, we have considered the fishing-based threats such as boat traffic, fishing gears and intensive fishing practices) on the dugong population present in this region. We observed that the existing Gulf of Mannar Marine National Park entirely falls under high risk for the existing dugong population.

In the GoK study site, the region of highly suitable habitat for dugongs (Fig. 2G) is entirely threatened by high risk. While the Gulf of Kutch Marine National Park is under moderate risk along the southern coast, the boat-based risk assessment highlights that the entire region of high and moderately suitable habitats is under a high-risk zone (Fig. 5). The low-suitability regions are threatened with moderate risk.

The overlap of high-risk regions, identified through this study with the existing protected area cover, varies across the study sites. Only 5.5% (26.16 sq. km out of 478.59 sq. km) of the high-risk region is protected in the ANI. About 28% of the high-risk region is protected in the PB-GoM region, where 1708.97 sq km is outside the current protected areas. In Gujarat, the existing Gulf of Kutch Marine National Park and other marine and coastal protected areas cover around 37% of the estimated high-risk area in this study (Fig. 3). All these high-risk areas, can be categorized as CDHs, including the area currently under protection and the predicted area beyond the MPA network.

## Discussion

Identifying suitable habitats and mapping risk is crucial to prioritize conservation actions^33^. In this study, we identified intensive-use areas by dugongs in highly restricted areas of their distribution along the Indian coast and pin-pointed high-risk zones to help inform conservation management. Through interview surveys of fisherfolk, we obtained information on a large temporal and spatial scale covering the entire extent of dugong distribution in India. The majority of data used in this study was from opportunistic sightings or secondary sources (fisher interviews, dugong volunteer network); SDM with presence-only data was employed, along with a combination of habitat suitability analysis and risk assessment to highlight areas of high risk for dugongs in its entire distribution range along the Indian coastline.

Seagrass habitats are declining worldwide^38^, necessitating country-wide assessments^39^ to facilitate conservation action. The availability of healthy seagrass meadows is considered one of the most important determinants of dugong occurrence and habitat use^40^. Dugongs, in turn, replenish seagrass meadows through constant grazing, helping nutrient cycling from sediments^7,41^. Through seagrass suitability analysis, we identified suitable seagrass habitats along most of the Andaman Islands (except parts of the northern coast), the whole PB-GoM region and the south-western coast of GoK. These areas, presumably the last remaining dugong habitats along most of the Indian subcontinent^13,26,42–45^, thus assume critical importance. Our current study considers only a single annual seagrass distribution layer for the dugong suitability analysis. The change in the contribution of seagrass across seasons indicates that other parameters influence the population over different times of the year (Table 1). However, this change in seagrass contribution across seasons highlights the importance of studying seasonal seagrass availability (density, composition, biomass, etc.) at all CDHs. Understanding the potential expanse of the seasonal seagrass habitats would also ignite efforts to assess nutrient profiling, seasonal seagrass biomass availability, carbon sequestration and seagrass-associated micro/macrofauna across spatial scales^40^.

Seasonal habitat suitability assessment paves the way for ecological interpretation in terms of migration, foraging, and reproduction^46^ and developing conservation strategies^47^. We found high suitable areas for dugong distribution to be restricted in pockets along the ANI coast even though the seagrass presence is predicted along the entire coastline of the region. The patches identified as high/moderate suitable and high-risk regions for dugong distribution show concurrence with the occupancy probability derived during previous studies^21^. Seagrass presence, depth, and distance from shore explained the habitat suitability of dugongs in the ANI region. Although the similarity correlation matrix (Supplementary Table S4 online) suggests negligible differences in suitable sites between seasons, there are considerable differences in the percentage contribution of variables between the seasons. This suggests the need to assess the seasonal distribution of dugongs^23^ and fine-scale mapping^35^ in high suitable but less surveyed areas (such as Little Andaman and central Nicobar). In the PB-GoM region, we observed stark seasonal differences in high suitable areas for dugongs. During pre-monsoon season, north Palk Bay, a gradually sloping sheltered embayment, appears to be a conducive habitat for dugongs. Monsoon prediction shows moderate suitability for the entire coastline, whereas high suitable areas shift to GoM during post-monsoon. Seasonal changes in habitat suitability, attributed to biotic (seagrass presence), topographic (slope) and environmental (sea surface temperature) factors, need further investigation. In the GoK region, seagrass presence and low organic turbidity) support clear shallow waters as preferred regions by dugongs^48^. With a relict dugong population in the GoK^26^, fine-scale mapping of seasonal dugong occurrence and seagrass distribution is required to address data deficiency in the entire region.

Dugongs are vulnerable to accidental entanglement in fishing nets and boat strikes as they surface regularly to breathe and, due to their slow movement, cause mortalities or life-threatening injuries^49^. Bycatch in fishing nets is a major threat to dugongs, whereas hunting for meat is also reported in some areas^50^. Our analysis shows that highly suitable dugong habitats are also subjected to high fishing pressure. This includes the eastern coast of the Andaman Islands, north Palk Bay, and the entire GoM, including the GoM Marine National Park and south-western GoK as high-risk areas. At ANI, despite many protected areas (nine National Parks, including two Marine National Parks and 94 Wildlife Sanctuaries) with part coastal cover, most areas identified as suitable for dugong distribution remain unprotected. CDHs extend beyond these protected areas, particularly in Aerial Bay, Diglipur, Little Andaman and Central Nicobar. Ground validation of risks in the north Andaman region suggests intensive monitoring is required in areas outside active protection.

Dugongs display both short-range (< 15 km) and long-range (> 15 km, up to 560 km) movement^8^. Our results indicate a high possibility of seasonal migration of dugongs in PB-GoM, given a considerable shift in suitability between seasons. It also points towards a potential movement across transboundary seagrass habitats along Sri Lankan coasts, particularly during the post-monsoon season. Additionally, the spatial extent and density of seagrass meadows in Indian waters change with season and anthropogenic activities^51^, which may facilitate the seasonal movements of dugongs. Longer migrations have been documented in areas of seagrass habitat loss with implications on dugong genetic diversity and their dietary preferences^33,52^. However, to validate long-distance movements, real-time tracking, or photographic capture-based studies (for example, using drones^53^) along with fine-scale environmental data collection is strongly recommended^54^.

**Figure 6:**
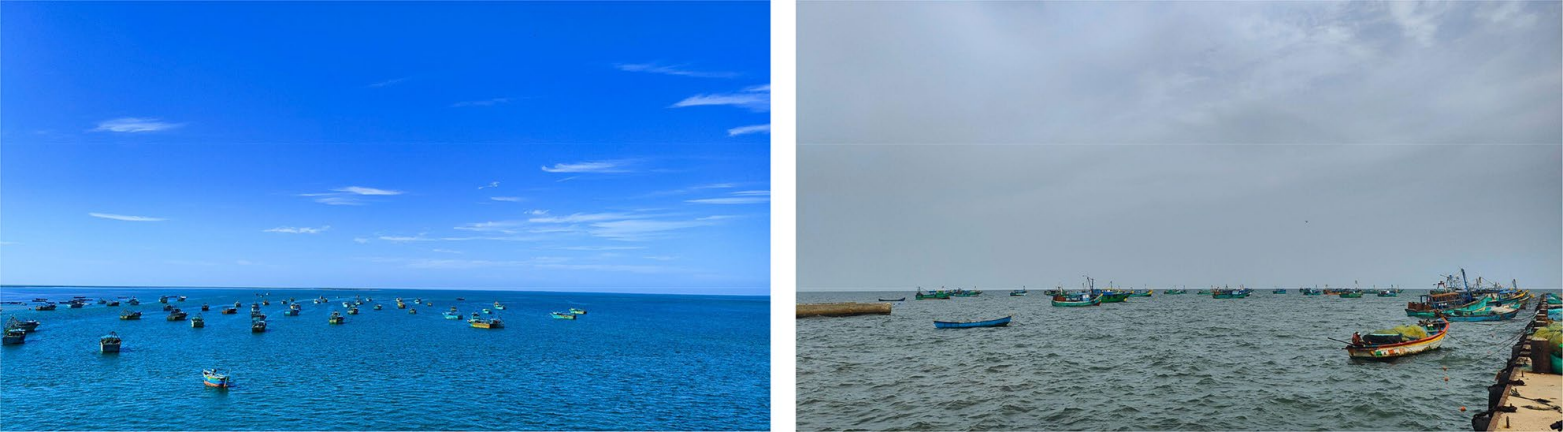
On-field photographs from Palk Bay to Gulf of Mannar, Tamil Nadu, showing a number of trawl boats.

With the influx of large, mechanized vessels and use of destructive fishing practices (such as bottom trawling) (Fig. 6), increased loss of seagrass habitats will have a dual negative impact, i.e., on fisheries of the region and on the seagrass-dependent dugong populations. The area estimated outside the existing protected area cover was about 95% for ANI, 72% for PB-GoM and 63% for the GoK region. Most of these areas are also intensive fishing grounds supporting millions of livelihoods. Effective management of these high-risk, high-priority areas is recommended, using a participatory approach to care for the fishing communities' needs through sustainable practices. Incorporating participatory management approaches (for example, community-supported space–time closures for high-suitability habitats in particular seasons) would require upscaling of community-led initiatives (such as Friends of Dugong Network^55^) and government-supported incentivization programs to reduce pressure on dugong habitats. Even introducing other participatory regulatory mechanisms such as Community Conserved Areas (CCAs) or Other Effective area-based Conservation Measures (OECMs) could be considered given the potential of the region to support high-value biodiversity, maintain the integrity of critical ecosystems (such as seagrass and coral reefs) and help in retention of genetic diversity. It would also support the country's efforts to recover endangered species as well as contribute towards the Convention on Biological Diversity's (CBD) goals to achieve, '30 × 30', reverse biodiversity loss and safeguard the rights of people^15^.

Overall, identifying such synoptic-scale 'Critical Dugong Habitats' (CDHs) with a model-based approach provides significant information for a large mammalian grazer, underlining the utility of citizen science and secondary data in performing large-scale spatial ecological analysis. Most importantly, it suggests an addition to India's current MPA network in dugong inhabited areas to contribute to the country's efforts towards achieving the '30 × 30' global biodiversity target with equitable and effective management to derive positive conservation outcomes.

## Materials and methods

### Study sites

**Figure 7:**
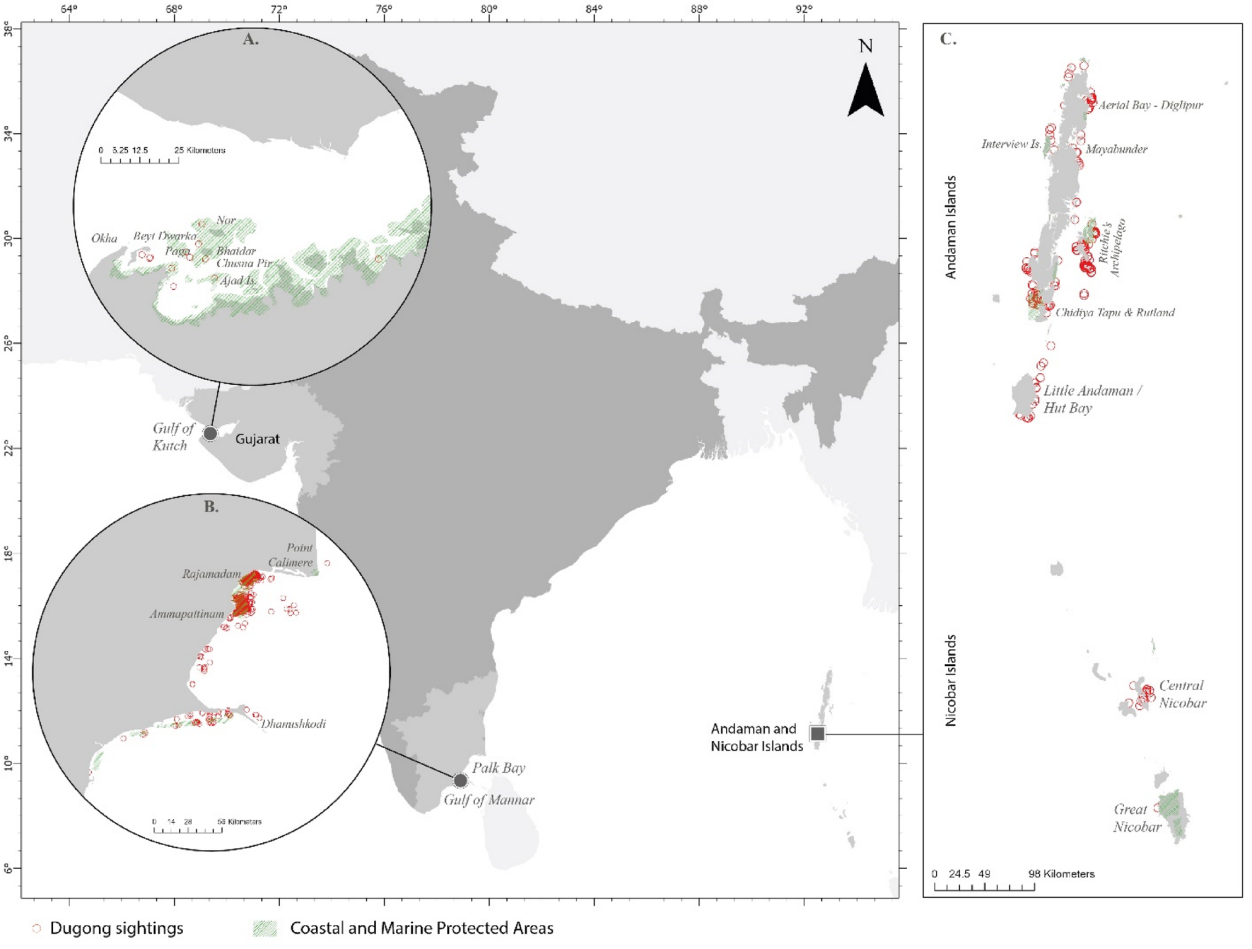
Study sites along the Indian coastline (**A**) Gulf of Kutch (GoK), Gujarat on the northwest coast in the Arabian Sea; (**B**) Andaman & Nicobar Islands (ANI)—offshore islands located in the Bay of Bengal to the south–east of peninsular India (**C**) Palk Bay and Gulf of Mannar (PB-GoM), Tamil Nadu to the south–east coast in the Bay of Bengal. The red circles represent dugong sighting locations between 2008 and 2021. The output maps were generated using ArcGIS Pro 3.2.1 software (https://www.esri.com/en-us/arcgis/products/arcgis-pro/overview).

In India, dugongs have restricted distribution in isolated pockets in Andaman & Nicobar Islands, Palk Bay- Gulf of Mannar, Tamil Nadu, and the Gulf of Kutch, Gujarat^43,56^ (Fig. 7).

These three regions are geographically disjunct (ANI are tropical oceanic islands, PB-GoM are enclosed bays on the south-east Indian coast, and GoK is a gulf located on the northwest Indian coast), with unique habitat structure, seagrass availability and extent, climatic patterns, and varying degree of anthropogenic threats.

The oceanic islands of Andaman and Nicobar (6° N–14° N and 92° E–94° E) hold high endemic biodiversity^57^. Over 80% of its land area (~ 8249 sq. km) is covered with tropical rainforests, with a long coastline (~ 1962 km) indented with several penetrating creeks^58^. Nine national parks (including two Marine National Parks), 96 wildlife sanctuaries, and one biosphere reserve cover about 20% of the total geographical area of the islands^59^. ANI consists of two major Island groups, the Andaman to the north and Nicobar groups of islands to the south, separated by the ten-degree channel. The islands receive rainfall mainly from May to September, with warm annual weather (mean annual temperature ~ 26.6 °C). ANI experiences high rainfall (1133–1725 mm) during southwest monsoon (May–September), followed by ~ post-monsoon (640–849 mm from October to December) and pre-monsoon showers (~ 443–527 mm from January to April)^60,61^. ANI host 12 seagrass species (dominated by *Halophila* sp., *Halodule* sp.^42^) with coverage of 12.239 km^2^ in the Andaman group and 17.194 km^2^ in the Nicobar group, respectively^62^, providing forage to a large number of dugongs (rough estimates suggest 44–81 dugongs^43^), with severe decline in dugong occupancy by ~ 60% over the last two decades^22^.

The PB-GoM region is part of the coastal waters of the southeastern state of Tamil Nadu. PB extends from Point Calimere in the north to Rameswaram in the south. GoM is designated from south of Dhanushkodi and extends till Kanyakumari at the tip of the Indian peninsula. PB is comparatively calmer than GoM due to obstructed wave action by the Sri Lankan coast, Rameswaram Island, and the Indian mainland. In contrast, GoM is exposed to swells from south to southwest^63^. Hence, PB provides a sheltered habitat, ideal for good growth of seagrass meadows, tidal flats, and mangroves^64^. GoM, on the other hand, is embedded with a string of island complexes (21 islands with two submerged), macroalgal beds (*Sargassum* spp., *Halimeda* spp., *Caulerpa* spp. and *Ulva* *reticulata*), seagrass meadows and patches of coral reefs. Gulf of Mannar Biosphere Reserve, the first biosphere reserve in south-east Asia, is known for its rich marine biodiversity (> 3600 documented species of flora and fauna)^65^. PB-GoM are economically important areas for demersal and pelagic fisheries^66^. The PB-GoM study sites host 14 seagrass species (*Cymodocea serrulata*, *Halophila ovalis*, *Halodule uninervis* etc.)^67^ spread over an area of ~ 398 km^2^^51^. PB-GoM also has the largest dugong population along the Indian coast and South Asia. Rough estimates peg the population to be ~ 150 individuals but with declining occupancy^13^.

The GoK, the largest gulf on the western Indian coast along the Arabian Sea, covers an area of 7350 km^2^ (457.92 km^2^ as Marine National Park and Sanctuary) with a cluster of 42 small coastal islands^68^. GoK encompasses the only marine national park along the west coast of India and the only marine sanctuary in the state of Gujarat. The region is located in the sub-tropical climatic zone and faces very high geo-morphological and climatic variation. It hosts eight seagrass species ^65^ (*Halophila ovalis*, *Halodule uninervis* etc.), covering ~ 23 km^2^^69^ and a relict dugong population of less than 10–15 individuals^26,43^.

### Habitat suitability

#### Data collection

We conducted intertidal and subtidal surveys between 2018 and 2021 using the line-intercept transect method^70^ to record seagrass presence in all three regions. We laid 50 m long transects perpendicular to the shoreline, where a 50 × 50 cm quadrat was placed after an interval of 5 m on the transect line. We also conducted boat-based surveys for subtidal seagrass meadows where diving was not possible, with appropriate visibility (~ 3–5 m) measured with a Secchi disk. We used the drop-down quadrate method, with a GoPro Hero 6 camera attached at the top of the quadrat frame^71^ to record seagrass presence. In areas with low water transparency (visibility < depth), we used Van-Veen grabs (such as in near-shore areas of PB-GoM and GoK). All locations were recorded using a Garmin eTrex 30 × handheld GPS unit.

We also extracted available seagrass polygons for our study areas from^72^. A multi-point file was generated using the 'polygon to point' conversion tool in ArcMap v.10.8. The seagrass locations obtained from the model were supplemented with the data from subtidal and intertidal surveys.

Historical information on dugong occurrences was curated from the questionnaire surveys of fisherfolk conducted between 2012 and 2013 (^13^; GoK = 8, ANI = 336 and PB-GoM = 263) and between 2018 and 2021 (^73^; GoK = 8, ANI = 226), following a standardized CMS-UNEP Dugong questionnaire. The survey was based on the revised protocols developed by the Project GLoBAL Rapid Bycatch Assessment (http://bycatch.env.duke.edu/) but also drew on protocols developed at the Phuket Marine Biological Center (Thailand), at San Francisco State University (USA) and at James Cook University (Australia)^74^. Verbal consent was obtained from every interviewee upon informing them of the purpose of the data collection.

Occurrence records of 45 locations by the GEER foundation in the GoK region were also utilized^43^. We obtained dugong sighting locations from a volunteer network comprising representatives from the fisher community, the Indian Navy, the Indian Coast Guard, and State Forest Departments at ANI (n = 63) and PB-GoM (n = 245). A few direct sighting records were added from primary field data obtained from boat-based surveys (n = 6 at ANI), drone-based surveys (n = 3 at ANI) and indirect presence from feeding trails (n = 3 at GoK). Eventually, after screening the metadata of occurrence information for locations, dates and positional inaccuracies, composite occurrence records (n = 64 at GoK, n = 634 at ANI and n = 508 at PB-GoM) were curated. Site-wise dugong sightings were further segregated for monthly occurrences, which were eventually categorized into seasonal data sets (Supplementary Fig. S7 online).

#### Developing a prediction-based model for dugong distribution

We ran three prediction models for pre-monsoon, monsoon, and post-monsoon seasons, respectively. All point locations were spatially rarefied using SDMtoolbox v.2.4 (available from www.sdmtoolbox.org)^75^ on ArcMap v.10.8 to reduce the negative influence of spatial autocorrelation on the models^76^. Seasonal segregation of sighting data was not appropriate for the GoK due to low data availability after bias correction. After data cleaning, dugong presence locations of ANI (pre-monsoon = 54, monsoon = 60 and post-monsoon = 56 points), PB-GoM (pre-monsoon = 99, monsoon = 18 and post-monsoon = 107 points) and GoK (n = 13 points) were retrieved. Southwest monsoon (SWM) winds determine monsoons in ANI, whereas Tamil Nadu experiences retreating monsoons caused by northeast monsoon (NEM) winds.

The seasons were site-specific, based on the monsoon report published by the India Meteorological Department (Supplementary Table S8 online).

#### Selection of environmental variables

To model the suitability of seagrasses, we selected fourteen abiotic layers to determine the seagrass distribution^37,72,77^. The resultant seagrass layer was further used to predict dugong habitat suitability^78^ along with five other abiotic layers (Table 1). Explanations behind choosing the variables are given in supplementary table S9 online as per data available at different sites. Given the data limitation from the GoK, annual habitat suitability was carried out with additional variable layers to compensate for seasonal variations. In this case, seven abiotic and one biotic variable were considered (Table 1).

We first checked the predictors for data availability, and then multicollinearity was reviewed using ENMTools v.1.0.5^79^ and reshape2 v.1.4.4 packages in R^80^. All predictor variables for seagrass and dugong suitability modelling were pre-processed and interpolated at a spatial resolution of 1 km using ArcMap v.10.8. We further resampled the layers to a similar extent using 'ENMTools v.1.0.6' and 'raster v.3.5–21' packages in RStudio v.2021.09.1.

#### Suitability modelling

We used MaxEnt v.3.4.4 software for seagrass and dugong modelling due to its utility in limited presence-only data^33,54,81^. For modelling seagrasses, we used the MaxEnt settings from^72^.

In the case of dugong suitability, the best possible MaxEnt settings combination was assessed using the ENMeval v2.0.1 package in R-Software^82^. We ran ENMeval for each season for ANI and PB-GoM and once for GoK using a random k-fold data partitioning technique (for > 50 occurrence points) and by jack-knifing (for < 50 data points)^83^. We selected the MaxEnt settings with the least Akaike Information Criterion (AIC) value for final runs (Supplementary Table S10 online).

All runs were performed using a bias file with the sighting data to avoid sampling biases^84,85^. The site and season-specific bias files were created using the kernel density function in R^86^.

In the case of both seagrass and dugong MaxEnt outputs, we selected 'logistic' as the output format, and the model accuracy was determined by the Area Under Curve (AUC) value. Final output maps were prepared using ArcMap 10.8. Response curves of different variables for ANI and PB-GoM, Tamil Nadu, are provided as supplementary Figs. S11, S12 online, respectively. For GoK-Gujarat, variable response curves are presented in the supplementary Fig. S13 online.

Final outputs were divided into four quarters of prediction probability of values 0–0.25 (unsuitable), 0.25–0.5 (low suitability), 0.5–0.75 (moderately suitable) and 0.75–1 (highly suitable) region, respectively. We also carried out a Niche Similarity Analysis to understand if the season-wise suitability of two sites (i.e., PB-GoM and ANI) are significantly different from each other, using I-statistics^87^ in ENM tools v.1.3^88^.

#### Classifying fishing pressure

We used the fishing pressure layer from^17^. This layer was created as grid-based polygons (n = 894) at a spatial resolution of 10 km. Covariates used in creating the layer were fishing months, motor type and power and gear type. The layer was interpolated to 1 km spatial resolution using ArcMap v.10.8.

#### Risk assessment

**Figure 8:**
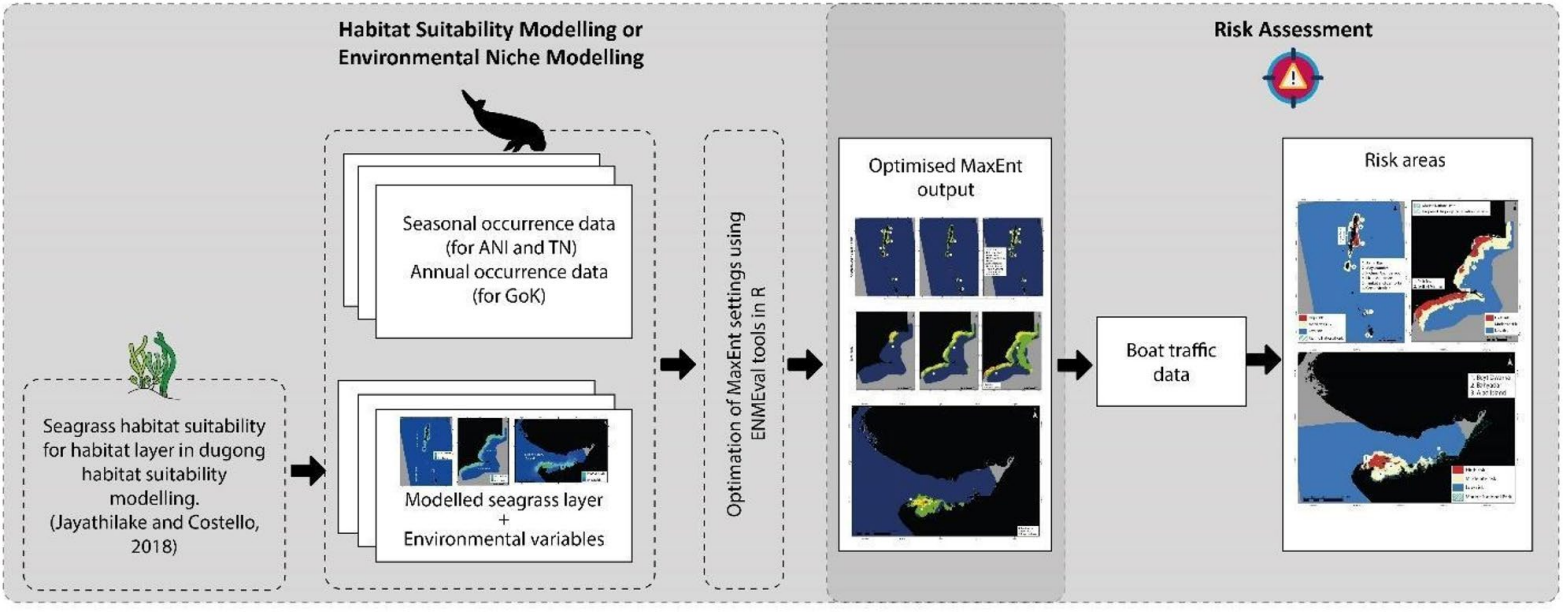
Graphical flowchart to show the methods used in detail to elucidate Critical Dugong Habitats in India.

Seasonal suitability layers were combined into one layer for each site, using the raster calculator function in ArcMap v.10.8. We multiplied the combined suitability layer with the fishing pressure layer to identify high-risk areas. The risk assessment layers were categorized by manually segregating the raster classification from 0 to 100%, with 100–51 classified as high risk, 50–26 as moderate risk and 25–0 as low risk on ArcMap v.10.8. These high-risk areas (over 50% risk) with high habitat suitability (over 0.75 prediction probability) were classified as Critical Dugong Habitats. A flowchart of the detailed methodology has been given in Fig. 8.

Further, we conducted boat survey-based threat mapping (fishing gear, vessel number and types) in representative high-risk areas from the three identified sites. This included Aerial Bay-Diglipur and Mayabunder area from North Andaman; Rani Jhansi Marine National Park (RJMNP, Ritchies' Archipelago) from South Andaman; Bhaidar, Ajad, Chushna Pir, Nor, Beyt Dwarka islands and Paga reef from GoK, Gujarat and coastal waters from Rajamadam to Ammapattinam in north Palk Bay region of PB-GoM. We scanned for boats and fishing gear for 10 min at the centroids of randomly selected 2 × 2 km grids at these sites. These surveys were conducted between 2019 and 21 in ANI, 2020–21 in Tamil Nadu, and 2020–22 in Gujarat. We ran an Inverted Distance Weightage (IDW) analysis at 1 km spatial resolution (powers 0.001–10; interval − 0.01) to classify high, moderate, and low threat areas. This layer was overlaid on the dugong habitat suitability layer to cross-verify the risk assessment conducted using secondary data. Areas of high risk outside the existing MPAs along the Indian coast were quantified to estimate proportional areas without legal protection at all the study sites.

### Ethics approval and consent to participate

The study was approved by the Research Ethics Review Committee of the Wildlife Institute of India. All procedures performed complied with the relevant guidelines and regulations.

## Limitations

Our study derives the results and inferences based on the CMS-UNEP Dugong Questionnaire, which obtained information from opportunistic sightings or secondary sources through interview surveys of fisherfolk, dugong volunteer network and other stakeholders. Although this effort highlights the application of citizen science in understanding spatial distribution patterns of marine mammals, these datasets pose few uncertainties. The first is the opportunistic dugong occurrence data from the extensive informant network. The seasonal occurrence data is potentially limited by the spatial and temporal bias of the range of fishing operations in the categorized seasons. For the PB-GoM region, pre-monsoon occurrence data was majorly available from Palk Bay. Supplementary Figure S6 online emphasizes the occurrence frequency across the year and the high values during the pre-monsoon season. These biases were addressed by implementing bias correction^84–86^ as well as rarefying the occurrences^75^ to one observation per grid for our analysis.

### Supplementary Information

Supplementary Information.

## Data Availability

The datasets generated during and/or analyzed during the current study are available from the corresponding author upon reasonable request.
