# Supplementary material for: Spatial prioritization of dugong habitats in India can contribute towards achieving the 30 × 30 global biodiversity target
Source: Sci Rep. 2024 Jun 17;14:13984. doi: 10.1038/s41598-024-64760-8 (PMC11183059; doi:10.1038/s41598-024-64760-8)
Supplement: Supplementary file 1 — Supplementary Information. [file 41598_2024_64760_MOESM1_ESM.docx]

**Supplementary Fig. S1:** Increasing trend in output by volume from marine fishery in ANI, Tamil Nadu and Gujarat for a period of 5 years. The data is collated from state-wise output from Handbook of Fisheries Statistics-2022 report, Department of Fisheries, Ministry of Fisheries, Animal Husbandry & Dairying, Government of India. A dip in overall output for the year 2021 can be attributed to regulated fishing activities during Corona-virus lockdown.
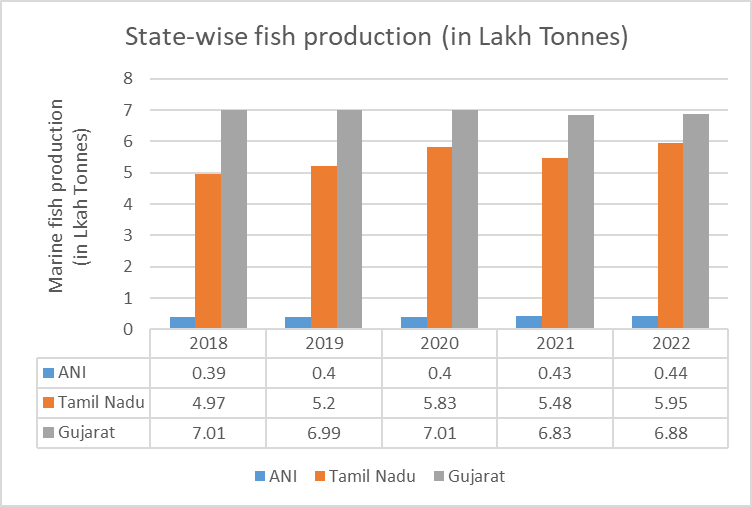


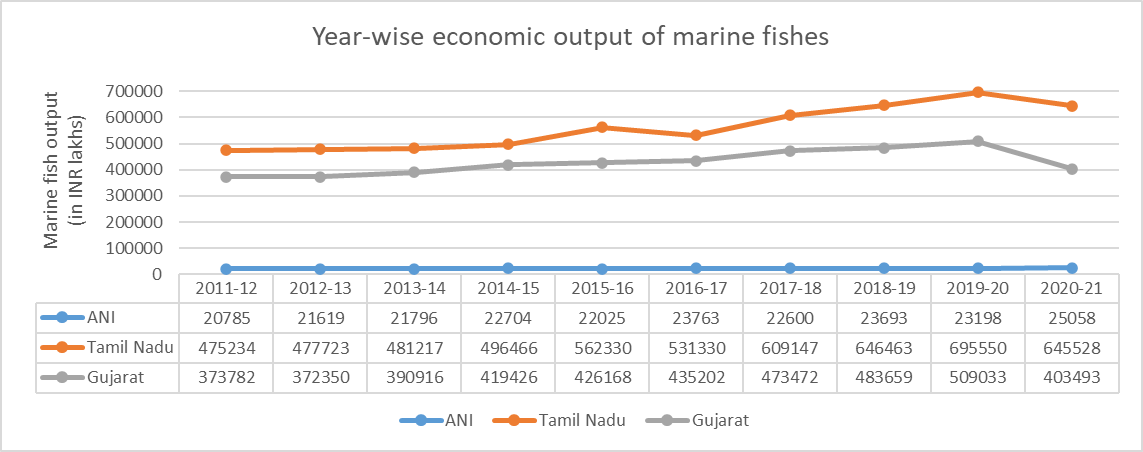


**Supplementary Fig. S2:** Increasing trend in economic output from marine fishery in ANI, Tamil Nadu and Gujarat for a period of 9 years. The data is collated from State-wise and item-wise value of output from agriculture, forestry and fishing Year: 2011-12 to 2020-21 with base year: 2011-12 (2023), National Statistical Office, Ministry of Statistics and Programme Implementation, Government of India. A dip in overall output for the year 2020-21 can be attributed to regulated fishing activities during Corona-virus lockdown.

**Supplementary Table. S3**: List of variables finalized for modelling seagrass in Andaman and Nicobar Islands, Palk Bay & Gulf of Mannar of Tamil Nadu, and Gulf of Kutch of Gujarat along with their contribution percentages. [‘-’ not used; Sources: * Global Marine Environmental Datasets (GMED) [35]]

| **Layers** | **Resolution** | **Andaman Nicobar Islands (ANI)** | **Palk Bay & Gulf of Mannar (PB-GoM)** | **Gulf of Kutch (GoK)** |
| --- | --- | --- | --- | --- |
| Depth (m) * | 30 arc sec | 71.8 | 0.6 | 8.9 |
| Slope (°) * | 5 arc min (~9.2 km) | 0.9 | 2.3 | 0 |
| Euphotic depth (m) * | 2.5 arc min (~4 km) | 5.2 | 2.6 | 6.2 |
| Distance from the shore (km) * | 5 arc min (~9.2 km) | 1.7 | 73.2 | 1.5 |
| Salinity (P.S.S.) * | 1° | 0.9 | 0.7 | - |
| pH* | 1° | 0.2 | - | 0 |
| Photosynthetically Active Radiation (P.A.R.) (Einstein/m2/day) * | 5 arc min (~9.2 km) | 0.6 | - | 7.8 |
| Sea Surface Wave Height (m) * | 5° | 12.5 | - | 0 |
| Surface current (m/s) * | 0.25° | 1.1 | 3.5 | 0.5 |
| Phosphate (ml/l) * | 1° | 2.9 | 1.1 | 40.8 |
| Nitrate (μmol/l) * | 1° | 0 | 1.1 | 5.7 |
| Diffuse Attenuation Coefficient (Kd) (m-1) * | 5 arc min (~9.2 km) | 0 | 2.6 | 2.4 |
| Sea Surface Temperature-mean (°C) * | 5 arc min (~9.2 km) | 0.5 | 1.7 | 26.2 |
| Sea Surface Temperature-max (°C) * | 5 arc min (~9.2 km) | 1.7 | 10.5 | 0.1 |

**Supplementary Table S4**: Information on I-statistics correlation matrix for niche similarity of different seasons in Andaman and Nicobar Islands and Palk Bay & Gulf of Mannar, Tamil Nadu.

| **ANI** | | | |
| --- | --- | --- | --- |
| **Seasons** | Pre-monsoon | Monsoon | Post-monsoon |
| Pre-monsoon | 1 | 0.9735568 | 0.9569629 |
| Monsoon | x | 1 | 0.9342551 |
| Post-monsoon | x | x | 1 |
| **PB-GoM, Tamil Nadu** | | | |
| **Seasons** | Pre-monsoon | Monsoon | Post-monsoon |
| Pre-monsoon | 1 | **0.7205054** | **0.7207523** |
| Monsoon | x | 1 | 0.937226 |
| Post-monsoon | x | x | 1 |


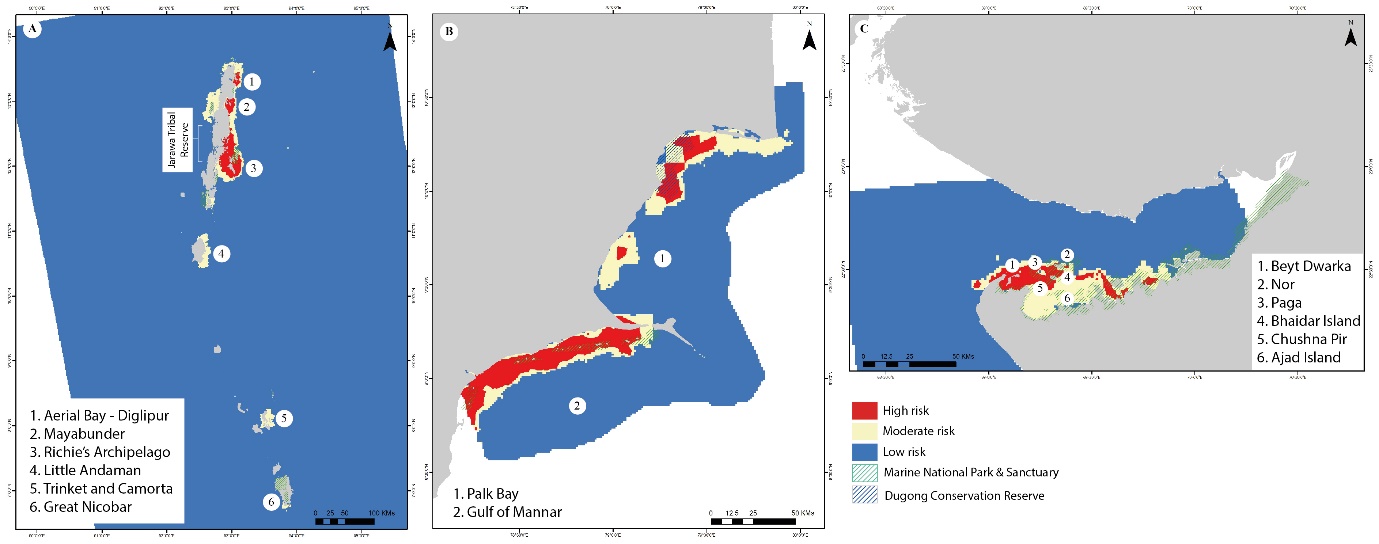

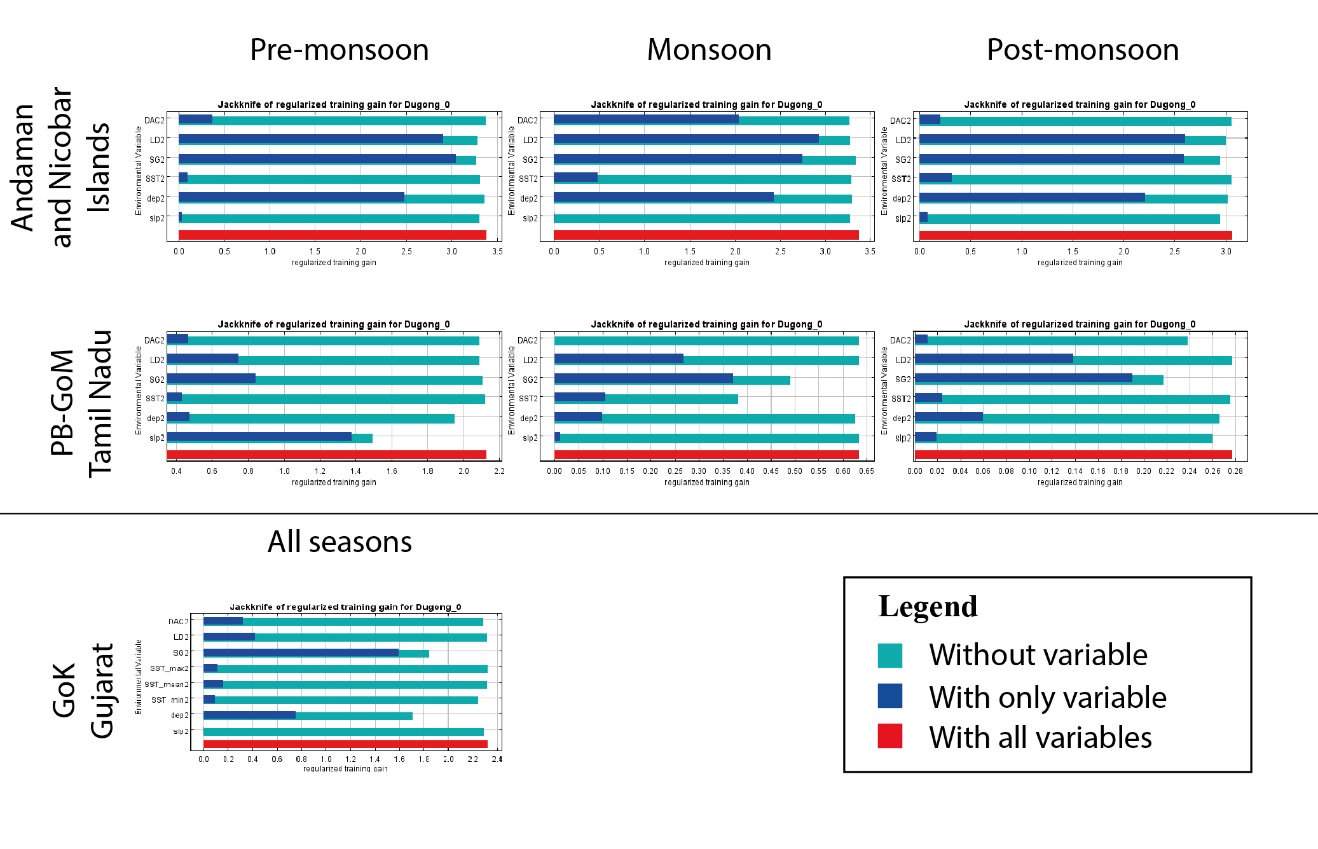
**Supplementary Figure S5**: Jackknife test results of variable importance using AUC on test data in predicting habitat suitability of dugongs in Andaman and Nicobar Islands, Palk Bay and Gulf of Mannar, Tamil Nadu and Gulf of Kutch, Gujarat.

**Supplementary Figure S6:** Risk predictions due to fishing pressure in A. Andaman and Nicobar Islands; B. Palk Bay and Gulf of Mannar, Tamil Nadu and C. Gulf of Kutch, Gujarat. The output maps were generated using ArcGIS Pro 3.2.1 software (https://www.esri.com/en-us/arcgis/products/arcgis-pro/overview).


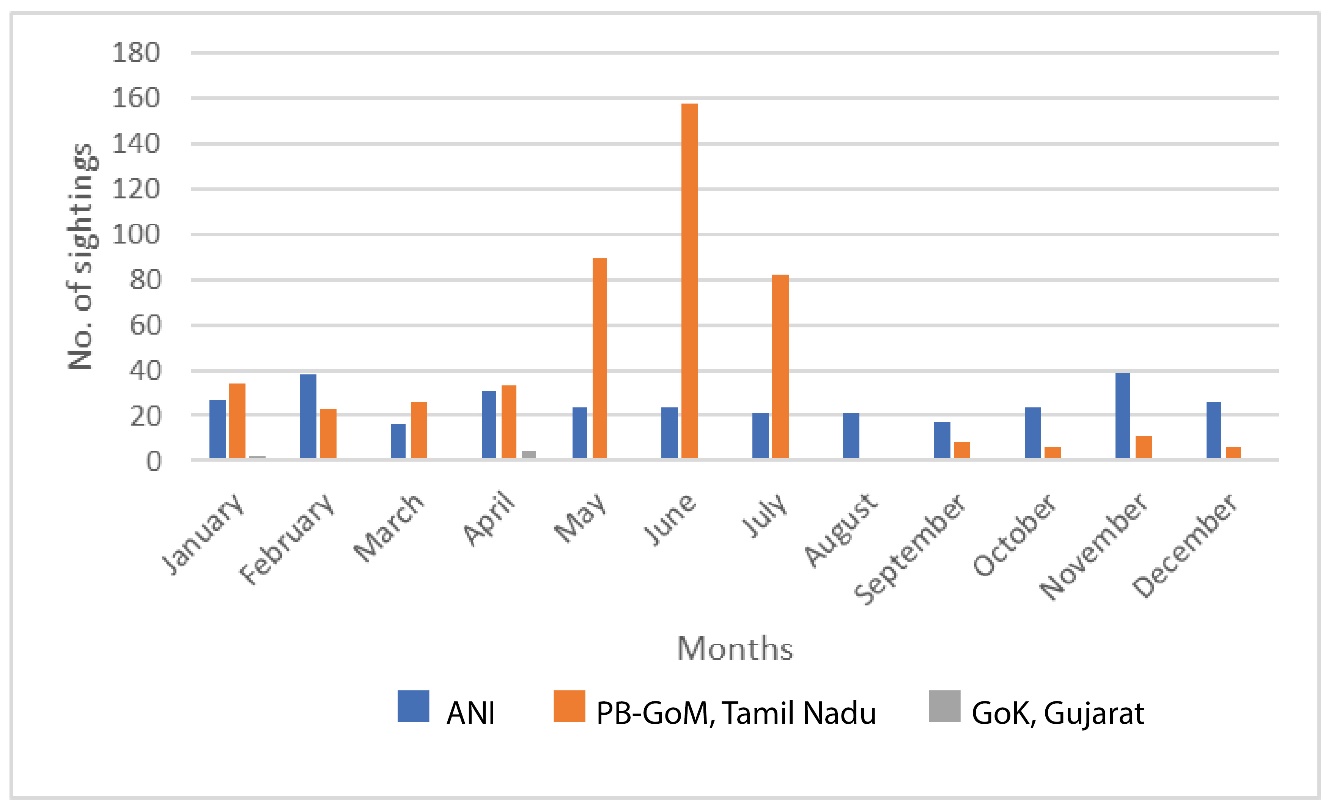


**Supplementary Figure S7**: Monthly occurrence of dugongs from all three study sites recorded between 2008-2021.

**Supplementary Table S8**: Classification of seasons with respect to monsoon in Andaman & Nicobar Islands, Palk Bay and Gulf of Mannar, Tamil Nadu.

| **Sites** | **Pre-monsoon** | **Monsoon** | **Post Monsoon** | **Reference** |
| --- | --- | --- | --- | --- |
| ANI (South-west Monsoon) | Jan-Apr | May-Sep | Oct-Dec | Bera et al., 2015; Sahu et al., 2013 |
| Tamil Nadu (North-east Monsoon) | Jun-Sep | Oct-Dec | Jan-May | IMD, 2020 |

**Supplementary Table S9:** Assumptions and relevance of explanatory variables used in this study with specific references.

| **Sl. No.** | **Layers** | **References** |
| --- | --- | --- |
| 1 | Depth (m) | Hashim, M., Ito, S., Numata, S., Hosaka, T., Hossain, M. S., Misbari, S., ... & Ahmad, S. (2017). Using fisher knowledge, mapping population, habitat suitability and risk for the conservation of dugongs in Johor Straits of Malaysia. *Marine Policy*, *78*, 18-25. |
| 2 | Slope (°) | As per ecological knowledge.  TN has undulating slopes favouring SG growth and hence dugongs whereas ANI has rugged bathymetry harbouring less number of dugongs.  Further, all the three geographical regions included in our study are of different coastal topography. |
| 3 | Distance from the shore (km) | Hashim, M., Ito, S., Numata, S., Hosaka, T., Hossain, M. S., Misbari, S., ... & Ahmad, S. (2017). Using fisher knowledge, mapping population, habitat suitability and risk for the conservation of dugongs in Johor Straits of Malaysia. *Marine Policy*, *78*, 18-25. |
| 4 | Monthly Diffuse Attenuation Coefficient (Kd) | As a proxy for turbidity. Turbidity as a predictor variable was suggested by-  Briscoe, D. K., Hiatt, S., Lewison, R., & Hines, E. (2014). Modelling habitat and bycatch risk for dugongs in Sabah, Malaysia. *Endangered Species Research*, *24*(3), 237-247. |
| 5 | Monthly Sea Surface Temperature-mean (°C) | Astudillo‐Scalia, Y., & de Albuquerque, F. S. (2020). The geography of high‐priority conservation areas for marine mammals. *Global ecology and biogeography*, *29*(12), 2097-2106. |
| 6 | Monthly Sea Surface Temperature-max (°C) | Astudillo‐Scalia, Y., & de Albuquerque, F. S. (2020). The geography of high‐priority conservation areas for marine mammals. *Global ecology and biogeography*, *29*(12), 2097-2106. |
| 7 | Monthly Sea Surface Temperature-min (°C) | Astudillo‐Scalia, Y., & de Albuquerque, F. S. (2020). The geography of high‐priority conservation areas for marine mammals. *Global ecology and biogeography*, *29*(12), 2097-2106. |
| 11 | Seagrass Presence | Seagrass being the major feed for dugongs. Modelled habitat suitability layer for seagrasses was used. |

**Supplementary Table S10**: Best fit maxent settings with least AIC values for modelling dugong distribution in the study sites of Andaman & Nicobar Islands, Palk Bay & Gulf of Mannar, Tamil Nadu and Gulf of Kutch, Gujarat (L = Linear, Q = Quadratic, H = Hinge, P = Product, T = Threshold, CV = Crossvalidate, BS = Bootstrap).

| **MaxEnt Settings** | **Andaman & Nicobar Islands** | | | **Palk Bay- Gulf of Mannar** | | | **Gulf of Kutch(n=13)** |
| --- | --- | --- | --- | --- | --- | --- | --- |
|  | **Pre-monsoon (n=54)** | **Monsoon (n=60)** | **Post-monsoon (n=56)** | **Pre-monsoon (n=99)** | **Monsoon (n=18)** | **Post-monsoon (n=107)** |  |
| Data partitioning | Random k-fold | Random k-fold | Random k-fold | Random k-fold | Jackknife | Random k-fold | Jackknife |
| Feature class | LQ | LQ | L | LQHPT | L | LQHPT | L |
| Output format | Logistic | Logistic | Logistic | Logistic | Logistic | Logistic | Logistic |
| Replicate run type | CV | CV | CV | CV | BS | CV | BS |
| No. of replicates | 10 | 10 | 10 | 10 | 18 | 10 | 13 |
| Regularisation Multiplier | 0.5 | 0.5 | 0.5 | 2 | 1.5 | 3 | 0.5 |
| Max. no. of background points | 10000 | 10000 | 10000 | 10000 | 10000 | 10000 | 5000 |
| Max. iterations | 1000 | 1000 | 1000 | 1000 | 1000 | 1000 | 1000 |
| Convergence threshold | 10^-5^ | 10^-5^ | 10^-5^ | 10^-5^ | 10^-5^ | 10^-5^ | 10^-5^ |


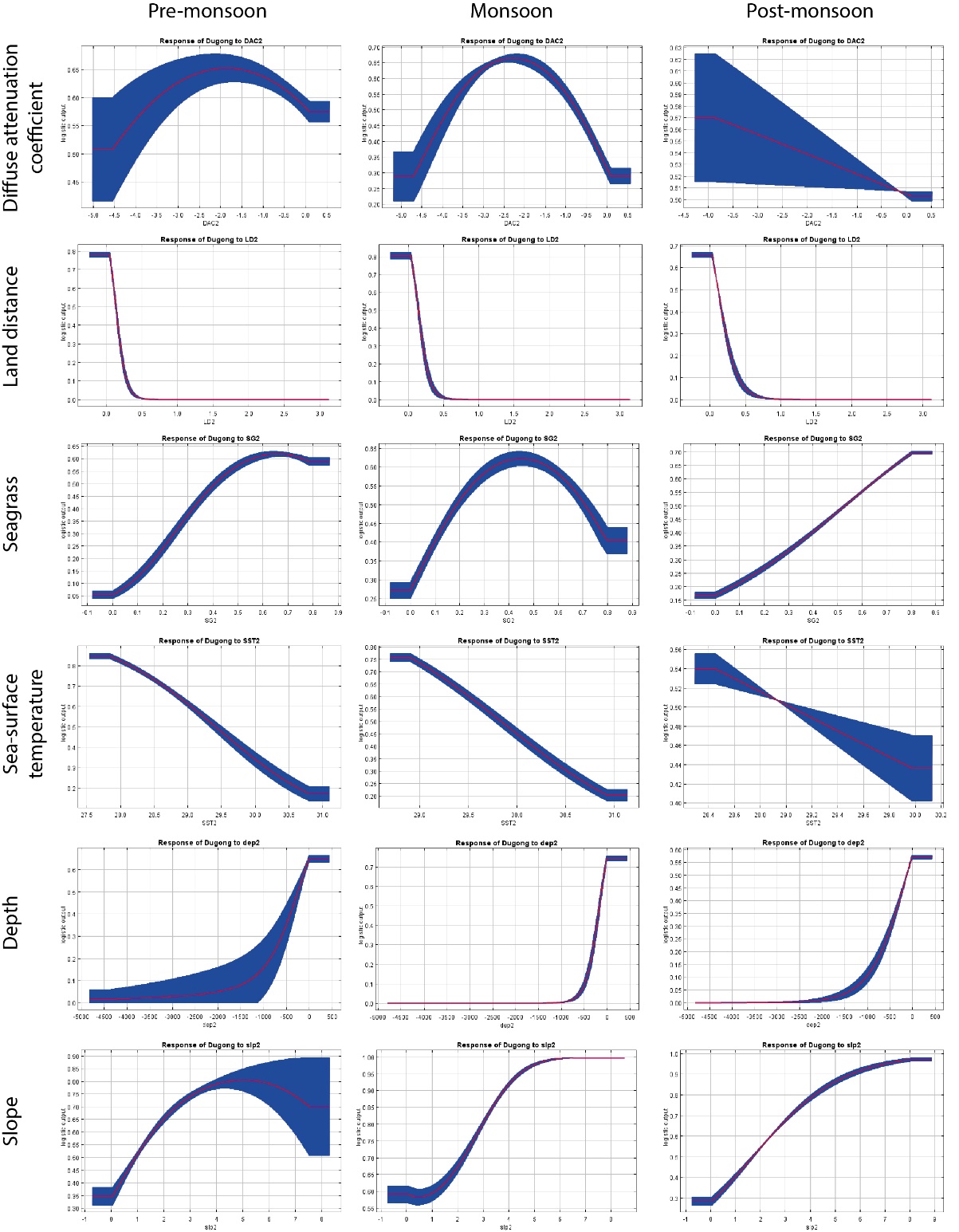
**Supplementary Figure S11**: Response curves of different variables for habitat suitability modelling for dugongs during pre-monsoon, monsoon and post-monsoon seasons in Andaman and Nicobar Islands.


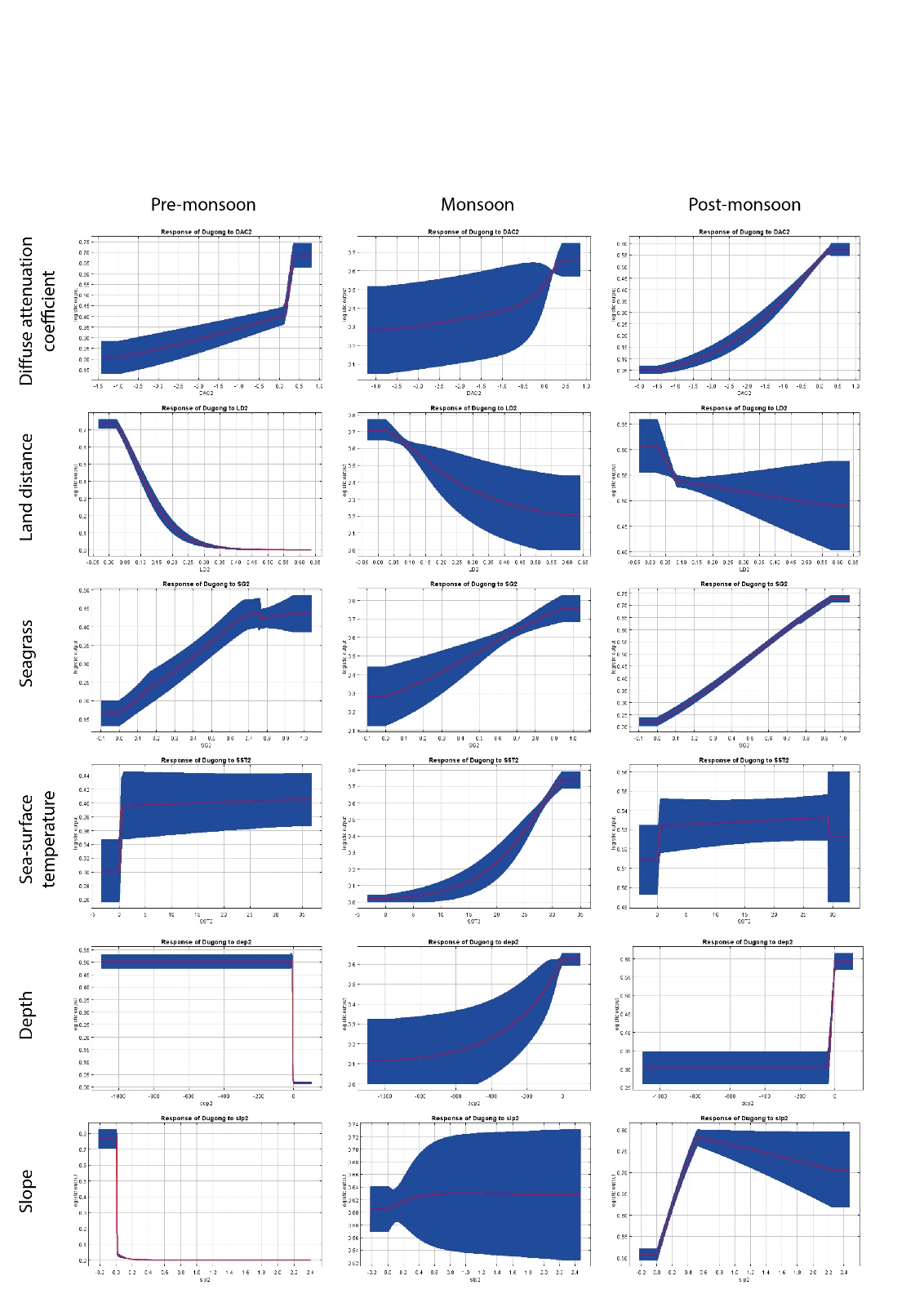
**Supplementary Figure S12**: Response curves of different variables for habitat suitability modelling for dugongs during pre-monsoon, monsoon and post-monsoon seasons in Palk Bay & Gulf of Mannar, Tamil Nadu.


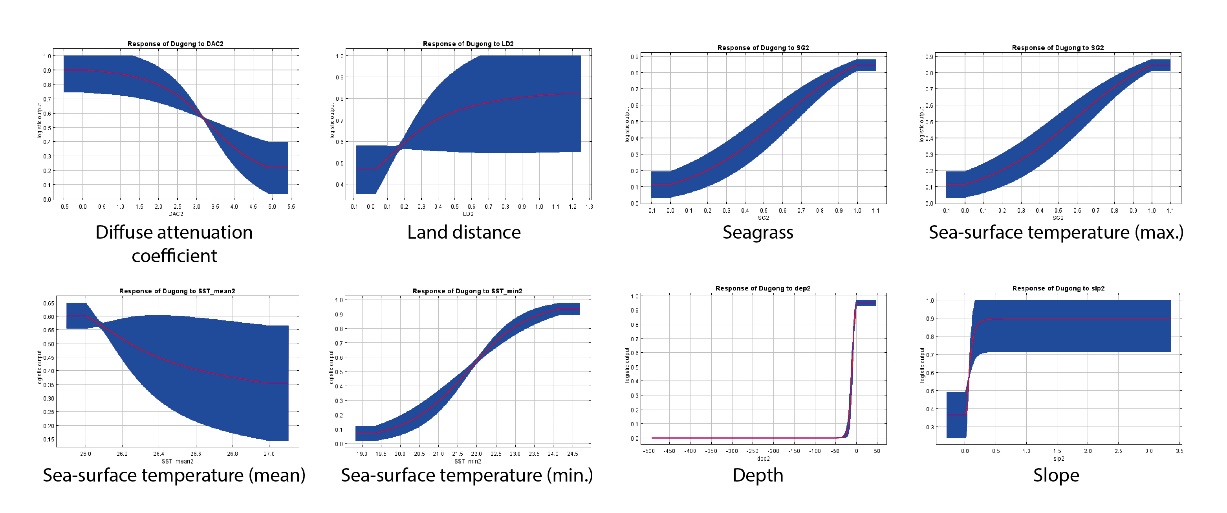
**Supplementary Figure S13**: Response curves of different variables for habitat suitability modelling for dugongs in Gulf of Kutch, Gujarat.
